# Can analyses of electronic patient records be independently and externally validated? Study 2—the effect of β-adrenoceptor blocker therapy on cancer survival: a retrospective cohort study

**DOI:** 10.1136/bmjopen-2014-007299

**Published:** 2015-04-13

**Authors:** David A Springate, Darren M Ashcroft, Evangelos Kontopantelis, Tim Doran, Ronan Ryan, David Reeves

**Affiliations:** 1 NIHR School for Primary Care Research, Centre for Primary Care, Institute of Population, Health, University of Manchester, Manchester, UK; 2 Centre for Biostatistics, Institute of Population Health, University of Manchester, Manchester, UK; 3 Centre for Pharmacoepidemiology and Drug Safety, Manchester Pharmacy School, University of Manchester, Manchester, UK; 4 Centre for Health Informatics, Institute of Population Health, University of Manchester, Manchester, UK; 5 Department of Health Sciences, University of York, York, UK; 6 Primary Care Clinical Sciences, School of Health and Population Sciences, University of Birmingham, Edgbaston, UK

**Keywords:** PRIMARY CARE, ONCOLOGY, STATISTICS & RESEARCH METHODS

## Abstract

**Objectives:**

To conduct a fully independent, external validation of a research study based on one electronic health record database using a different database sampling from the same population.

**Design:**

Retrospective cohort analysis of β-blocker therapy and all-cause mortality in patients with cancer.

**Setting:**

Two UK national primary care databases (PCDs): the Clinical Practice Research Datalink (CPRD) and Doctors’ Independent Network (DIN).

**Participants:**

CPRD data for 11 302 patients with cancer compared with published results from DIN for 3462 patients; study period January 1997 to December 2006.

**Primary and secondary outcome measures:**

All-cause mortality: overall; by treatment subgroup (β-blockers only, β-blockers plus other blood pressure lowering medicines (BPLM), other BPLMs only); and by cancer site.

**Results:**

Using CPRD, β-blocker use was not associated with mortality (HR=1.03, 95% CI 0.93 to 1.14, vs patients prescribed other BPLMs only), but DIN β-blocker users had significantly higher mortality (HR=1.18, 95% CI 1.04 to 1.33). However, these HRs were not statistically different (p=0.063), but did differ for patients on β-blockers alone (CPRD=0.94, 95% CI 0.82 to 1.07; DIN=1.37, 95% CI 1.16 to 1.61; p<0.001). Results for individual cancer sites differed by study, but only significantly for prostate and pancreas cancers. Results were robust under sensitivity analyses, but we could not be certain that mortality was identically defined in both databases.

**Conclusions:**

We found a complex pattern of similarities and differences between databases. Overall treatment effect estimates were not statistically different, adding to a growing body of evidence that different UK PCDs produce comparable effect estimates. However, individually the two studies lead to different conclusions regarding the safety of β-blockers and some subgroup effects differed significantly. Single studies using even internally well-validated databases do not guarantee generalisable results, especially for subgroups, and confirmatory studies using at least one other independent data source are strongly recommended.

**Strengths and limitations of this study**

- Drug effectiveness studies, applying the same analysis protocol to different electronic health record (EHR) databases, have typically compared EHRs covering different patient populations or replications, but have not been independently conducted. This paper reports on a fully independent validation of a published EHR-based study using a different EHR database sampling from the same underlying population.
- Despite purporting to cover the same general UK population, there were some notable demographic and clinical differences between the Clinical Practice Research Datalink and Doctors’ Independent Network cancer cohorts. Sensitivity analysis indicated that these had only a minimal effect on treatment effect estimates, but we were unable to account for a difference in mortality rates between the cohorts.
- The present study adds to evidence from our previous independent replication study and other non-independent replications, that the application of identical analytical methods to a variety of different UK primary care databases produces treatment effect estimates that are in most respects comparable. Nevertheless, we also find that single studies, even when based on these well-validated data sources, do not guarantee generalisable results.

## Introduction

Large-scale electronic health record databases (EHRs) are widely regarded as an important new tool for medical research. The major UK ‘primary care databases’ (PCDs) are some of the largest and most detailed sources of electronic patient data available, holding detailed long-term clinical data for many millions of patients. Researchers are increasingly using these resources^1^ which provide a means for researching questions in primary care that cannot feasibly be addressed by other means, including unintended consequences of drug interventions, where ethical considerations, the required numbers of patients, or length of follow-up can make a randomised controlled trials impractical.

Concerns remain, however, about the validity of studies based on such data, including uncertainties about data quality, data completeness and the potential for bias due to measured and unobserved confounders. Most work on EHR validity has focused on the accuracy or completeness of the individually recorded data values, such as consultation recording,^2^ disease diagnoses^3^ ^4^ and risk factors.^5–7^ Another approach for testing the validity of EHR-based studies is to compare the results to those obtained from equivalent investigations conducted on other independent data sets. Agreement of results helps to reassure that the findings do not depend on the source of the data, although agreement does not rule out the possibility that common factors, such as confounding by indication, may be influencing results based on both sources.

Studies that have taken this approach and applied the same design protocol to more than one database have at times produced findings that closely agree, but have more often yielded inconsistent and even contradictory results. The largest of these studies systematically examined heterogeneity in relative risk estimates for 53 drug–outcome pairs across 10 US databases (all with more than 1.5 million patients), while holding the analytical method constant.^8^ Around 30% of the drug–outcome pairs had effect estimates that ranged from a significantly decreased risk in some databases to a significantly increased risk in others; only 13% were consistent in direction and significance across all databases. However, there was wide variability between the data sets, which ranged from commercial insurance claims data to electronic health records, and from Medicare recipients to US veterans to privately insured citizens. Most other comparative studies have likewise been based on quite disparate databases, such as different countries,^9–13^ different geographical areas of the same country,^10^ ^11^ different patient populations within a country,^8^ or different kinds of databases (eg, administrative claims data and electronic health records^8^).

These studies make the reasons for the heterogeneity in results unclear: in particular, the extent to which variability in results is due to differences in data recording and quality between databases, to differences in demographics and health between the covered populations, or may even be a product of random processes and statistical artefacts. Untangling the factors driving heterogeneity of results is important for helping to identify which data sources and results can be given credence and therefore, be used to inform on health decisions and policy.^14^

To help address this issue, comparisons that apply identical methods to two or more independent databases sampling from the same underlying patient population are useful. By keeping the population and methods constant across databases, we can better determine the extent to which the database systems per se produce variability in the results. However, studies of this form are few and far between. Two replication studies using different UK PCDs reported closely corresponding results using different database sources;^15–17^ however, these replications were conducted by research groups instrumental in the creation and maintenance of the comparator PCD and hence, lacked independence. In a previous paper,^1^ we used the Clinical Practice Research Datalink (CPRD) database^18^ to conduct an exact and independent replication of a study, originally undertaken in the QResearch database,^19^ on the impact of statins on survival in patients with coronary heart disease.^20^ These databases have no practices in common and use data drawn from different practice electronic record systems (EMIS and VISION, respectively). Reassuringly, our results using CPRD were in all main respects identical to those found with QResearch, particularly for the main outcome of overall risk of death associated with statins, which was lower by 55% in CPRD compared with 53% in QResearch.

To further build the evidence base on the validity of studies conducted using UK PCDs, in this paper we report on our second independent replication of a PCD study, comparing results derived from CPRD with results from a previously published study that used another PCD—the Doctors’ Independent Network (DIN)^21^—that also does not overlap with CPRD in either practices or record system. The original study by Shah *et al*^22^ compared all-cause mortality in patients with a new diagnosis of solid cancer receiving β-blockers with mortality in similar patients receiving alternative antihypertensive medications. This represents a different clinical topic to those addressed by previous replications.

## Methods

The DIN is an anonymised database drawing data from over 300 general practices using Torex software, covering over three million patients since 1989.^21^ There is no overlap between the practices in DIN and those in CPRD. An additional feature that makes DIN appealing for present purposes is that it is built around a quite different philosophy of how the medical record should be structured. CPRD records consultation notes as a sequence of discrete episodes, essentially unconnected, whereas DIN is based around the concept of the Problem Oriented Medical Record (POMR), which treats the medical record as a series of discrete but interconnected problems, with prescriptions linked to diagnoses under problem headings.^23^

Data for 1998 for a subset of 142 DIN practices, that passed data quality control checks for that year, demonstrated very high comparability in age and gender structure to both CPRD and Office for National Statistics midyear population estimates,^21^ although the DIN practices are somewhat more likely to be located in Southern areas of the UK (I Carey, personal correspondence). Prescription records are similar^23^ ^24^ and good agreement has been reported for ischaemic heart disease and hay fever prevalence,^21^ and the recording of 30 common childhood conditions.^25^

As in our previous replication, we focused on studies of the effectiveness of medicinal interventions and after assessing the relevant studies that had been conducted in DIN, we chose to replicate an investigation into the effects of β-blocker treatment on cancer survival by Shah *et al*^22^. This study concerned quite a different patient group and class of drug than our previous replication, and a relatively small treatment effect as opposed to a large one. In addition, the topic under investigation was an incidental drug effect—suggested by earlier in vitro studies^26^—that has sparked a great deal of medical community interest and the related research activity is still ongoing.^27^ The results of this activity have been very mixed and often contradictory, with some studies finding a protective effect for β-blocker use in relation to mortality from breast cancer^28^ ^29^ and others finding no effect or a modestly increased risk for various cancers, including lung, breast and prostate,^30^ and substantially increased risk of developing more advanced colon cancer.^31^ Interpretation of this variation in results is not simple as there are many differences between the studies, including the types of β-blockers involved, which could influence the relationship to mortality.^32^

In spite of this complexity, for the purposes of this paper, we are primarily concerned with the findings of the particular study by Shah *et al*,^22^ whose DIN-based analysis produced some evidence for an increase in all-cause mortality in patients with cancer receiving β-blockers. The size of the effect across the total sample was small, but not insubstantial (an 18% increase in risk of death), with subgroup analyses suggesting that this reflected larger effects mostly confined to patients with pancreatic and prostate cancers, and to those on β-blockers without additional blood pressure lowering medicines (BPLM). In their paper, Shah *et al*^22^ acknowledge that they cannot easily explain these results, but conclude that their study does not support the hypothesis that β-blockers improve survival for common cancers.

Using CPRD, we replicated the methods of Shah *et al*^22^ as closely as possible, given the differences between the two databases. The methodological details provided in the published paper were not sufficient by themselves to allow a close replication to be conducted and we therefore obtained additional details from the authors. We requested purely factual information about the methods used and did not share any of our analyses or results. All of the methods described below, including the study period, variable specifications and analytical procedures, are exact replications of those used in the original study, unless indicated otherwise.

We selected all practices in CPRD that provided up to standard data (according to CPRD's designation for data meeting their internal quality standards) for the whole of the period from 1 January 1997 to 31 December 2006. Within these practices and period, we next identified all patients aged 40–85 years with a first diagnosis of a solid tumour of the breast, lung, stomach, oesophagus, colon, renal system, prostate or ovary, and with at least two prescriptions of an antihypertensive drug (β-blockers, ACE inhibitors, angiotensin receptor blockers, thiazides, calcium channel blockers, α-adrenoceptor blockers) in the year prior to diagnosis. We excluded patients with specific indications (coronary heart disease, heart failure, arrhythmias, stroke) or contraindications (chronic obstructive pulmonary disease, diabetes, asthma, renal disease) for antihypertensive medication that may impact on survival, prior to cancer diagnosis. Indications were determined using the Read code lists for the original study as provided to us by Shah *et al*.^22^ We then classified the remaining patients, according to exposure in the 1-year period prior to cancer diagnosis, into three groups: (1) β-blockers plus other BPLM; (2) β-blockers but no other BPLM; (3) other BPLM only (controls). All Read codes used in the study are available on the Clinical Codes repository at https://www.clinicalcodes.org^33^

We extracted data for these patients for 1 year prior to cancer diagnosis up to the end of 2007 or until the last recorded date for practices that stopped providing data before the end of 2007; this gave a maximum possible length of follow-up postdiagnosis of 10 years. We intentionally made no attempt to ‘improve’ on the analysis conducted by Shah *et al*^22^ as our specific aim was to determine whether the same results and conclusions would emerge from using identical methods on a different underlying data set.

### Analysis

The main outcome was all-cause mortality, identified through a record of deaths in the CPRD. Patients who left their practice during the follow-up period were treated as censored observations in the analysis. Analysis used a Cox proportional hazards model with adjustment for patient age (below 55, 55–65, 66–75, 76 years or older), gender, year of diagnosis, smoking status (current, ex-smoker, never smoked, not recorded, as recorded in the year prior to diagnosis), number of medications received in year prior to diagnosis, Regional Health Authority, and practice postcode Index of Multiple Deprivation.^34^ The only measure not defined in the same way as by Shah *et al*^22^ was deprivation, which was at the patient level in DIN but in the practice in CPRD (see below).

Again following Shah *et al*,^22^ we conducted an analysis for each cancer site separately and then combined across sites using a DerSimonian-Laird random effects meta-analysis. Analyses were undertaken to compare all patients receiving β-blockers with the controls and also for patients subdivided into those receiving and those not receiving additional BPLM. We further undertook analyses for non-selective β-blockers only. Missing patient demographic information was dealt with by adding a category ‘missing’ to the levels of the variables (table 1). For all event variables (ie, cancer diagnoses, prescriptions, deaths), absence of a relevant code in CPRD was taken to indicate no such event.

**Table 1:** Comparison of CPRD and DIN patient cohorts (%, (n))

Variable	CPRD (n=11 302)	DIN (n=3462)
Age at diagnosis (years)		
18–55	5.3% (602)	NA
56–65	23.3% (2631)	NA
66–75	37.4% (4228)	NA
75 and above	33.9% (3841)	NA
Gender		
Male	55.3% (6247)	47.4% (1641)
Female	44.7% (5055)	52.6% (1821)
Smoking		
Current	14.7% (1665)	19.1% (661)
Ex-smoker	43.9% (4962)	27.2% (941)
Never smoked	34.2% (3864)	51.8% (1792)
Missing	7.2% (811)	1.9% (68)
Deprivation (IMD 2004 quintiles)*		
1 (most deprived)	17.8% (2012)	9.6% (333)
2	19.9% (2253)	14.9% (517)
3	21% (2375)	19.2% (664)
4	23.1% (2613)	22.0% (760)
5 (least deprived)	18.1% (2049)	26.4% (915)
Missing	0% (0)	7.9% (273)
Year of diagnosis		
1997–1998	5.8% (658)	12.1% (420)
1999–2000	8.8% (996)	15.8% (546)
2001–2002	16.4% (1856)	20.6% (714)
2003–2004	29.2% (3303)	25.1% (870)
2005–2006	39.7% (4489)	26.3% (912)
Medications (N)		
0–4	14.9% (1681)	16.9% (586)
5–9	29.4% (3319)	38.1% (1318)
10–14	28.2% (3188)	24.4% (845)
15–19	8.5% (956)	9.6% (332)
20 and above	19.1% (2158)	7.8% (269)
Missing	0% (0)	3.2 (112)
Prescribed β-blocker		
No	64.3% (7272)	59.4% (2057)
Yes	35.7% (4030)	40.6% (1405)
Type of β-blocker		
Atenolol	73.0% (2943)	75.2% (1057)
Propranolol	11.0% (443)	12.8% (180)
Other β-blocker	16.0% (644)	12.0% (168)

*Based on patient postcode for DIN and practice postcode for CPRD.

CPRD, Clinical Practice Research Datalink; DIN, Doctors’ Independent Network; NA, not available.

To make direct comparisons of the overall and cancer-specific treatment effect estimates (HRs) from CPRD with those reported by Shah *et al*^22^ for DIN, we used a Wald test, computed as the difference between the natural logs of the two HRs divided by the SE (derived from the logged CI limits) and tested as a Z-score.^35^

All analyses were performed using R V.3.0.2.^36^ In line with Shah *et al*,^22^ we used an α level for statistical significance of 5% throughout.

### Sensitivity analyses

We repeated the sensitivity analysis in the original study by excluding patients with less than 1-year survival after cancer diagnosis. The definitions of death and deprivation differed between databases and to assess sensitivity to this we repeated the analyses, with the CPRD sample restricted to practices for which linkages to patient-level IMD scores and Office for National Statistics (ONS) official death dates were available (58% of practices covering 60% of patients).

We observed notable differences between the cohorts regarding cancer site prevalence rates, area deprivation, year of diagnosis and patient gender (table 1). Some of these differences, particularly year of diagnosis, are likely related to a considerable increase in the number of practices in the CPRD over the time of the study, compared with DIN (see online supplementary table S1). To examine the sensitivity of our results to these database differences, we performed a sensitivity analysis on samples of the CPRD data that matched the make up of the DIN cohort in key aspects.

To do this, we used an iterative proportional fitting (IPF)^37^ algorithm, a method for matching marginal distributions that does not assume independence between the matching variables. The method is described in detail in online supplementary file 1. The matching variables were cancer site prevalence, year of diagnosis and area deprivation. The algorithm calculated selection probabilities (weights) for each patient in the CPRD data that were used to draw 10 000 weighted bootstrap samples (ie, samples with replacement). Each sample was analysed and the results combined to obtain overall estimates of effect (the median HR) and 95% CIs (2.5 and 97.5 centiles). The IPF algorithm produced an excellent level of agreement on all three matching variables and also corrected the imbalance on gender, but not smoking status (see online supplementary table S2). On average, each bootstrap sample consisted of 1352 patients on β-blockers and 2753 on other BPLMs. We also ran an analysis adjusting for the clustering of patients within practices, which Shah *et al*^22^ performed but did not report as it made no difference to the results (personal correspondence).

## Results

### Comparison of patient cohorts

Table 1 compares the patient cohorts from CPRD and DIN on key measures. As expected, given the greater number of practices in CPRD, the total sample was much larger (11 302 from 582 practices vs 3462 from 171 practices). Patients in the CPRD cohort were more likely to be male (55% vs 47%), to be an ex-smoker (44% vs 27%), to live in a more deprived area (38% vs 25% in the 2 most deprived quintiles), and to have been recently diagnosed with cancer (69% vs 51% since 2003). CPRD patients were also more likely to have a higher number of recorded medications (56% vs 44% on 10 or more medications), though the rate of β-blocker prescription in CPRD was a little lower (36% vs 41%). The breakdown of types of β-blockers used was similar in both cohorts, with around three-quarters of patients on atenolol (based on their last prescription before cancer diagnosis): this minimises the risk that the comparison might be affected by differential associations between mortality and β-blocker type.^32^

There was a much higher rate of prostate cancer in the CPRD cohort (table 2: 33% vs 22%), but lower rates of ovarian and renal cancers—though absolute numbers of these were low in both cohorts. Rates for other types of cancer were all similar. The overall mortality rate was also considerably higher in CPRD (table 2: 50% vs 42%), though median lengths of follow-up were similar (29 vs 30 months), as were survival rates 1 year postdiagnosis (78% vs 74%). The disparity in overall mortality rates was not resolved by matching the CPRD and DIN samples (see online supplementary table S2: 55% vs 42%), nor was it resolved after further matching the samples on smoking status (51% vs 42%); hence, disparity cannot be attributed to cohort differences in cancer rates, year of diagnosis or patient demographic factors. The overall mortality rate was also not reduced in the CPRD sensitivity data set restricted to patients with ONS deaths and patient level IMD scores (51%).

**Table 2:** Comparison of CPRD and DIN patient cohorts by exposure to BPLM in the year prior to cancer diagnosis

	All patients				Other BPLMs but no β-blockers				β-Blockers plus other BPLMs				β-Blockers only (β-blockers but no other BPLMs)			
	CPRD		DIN		CPRD		DIN		CPRD		DIN		CPRD		DIN	
	N	Per cent	N	Per cent	N	Per cent	N	Per cent	N	Per cent	N	Per cent	N	Per cent	N	Per cent
All patients	11 302	100	3462	100	7272	64.3	2056	59.4	2832	25.10	864	24.4	1198	10.6	542	15.7
Deaths	5754	50.1	1441	41.6	3748	51.6	846	41.2	1459	51.5	350	40.5	547	45.7	245	45.2
Alive at 1-year follow-up	8763	77.5	2576	74.4	5607	77.1	1541	75.0	2194	77.5	630	72.9	962	80.3	405	74.7
On non-selective β-blocker	685	6.06	239	7.5	NA	NA	NA	NA	359	12.7	71	8.2	326	27.2	167	30.8
Cancer sites																
Breast	2943	26.0	984	28.4	1746	24.0	554	26.9	794	28.0	240	27.8	403	33.6	194	35.8
Colon	1799	15.9	619	17.9	1104	15.2	354	17.2	476	16.8	162	18.7	219	18.3	103	18.9
Lung	1326	11.7	436	12.6	913	12.6	277	13.5	307	10.9	105	12.1	106	8.8	54	9.9
Oesophagus	434	3.8	159	4.6	257	3.5	95	4.6	116	4.1	44	5.1	61	5.1	20	3.7
Ovarian	203	1.8	148	4.3	124	1.7	76	3.7	45	1.6	43	5.0	34	2.8	29	5.4
Pancreas	376	3.3	140	4.0	222	3.1	83	4.0	111	4.0	34	3.9	43	3.6	23	4.2
Prostate	3748	33.2	759	21.9	2604	35.8	500	24.3	856	30.2	182	21.0	288	24.0	77	14.1
Renal	141	1.2	124	3.6	81	1.1	69	3.4	48	1.7	33	3.8	12	1.0	22	4.0
Stomach	332	2.9c	93	2.7	221	3.0	52	2.5	79	2.8	21	2.4	32	2.7	20	3.7

BPLMs, blood pressure lowering medicines; CPRD, Clinical Practice Research Datalink; DIN, Doctors’ Independent Network; NA, not applicable.

### Survival analysis

#### Overall mortality

**Figure 1:**
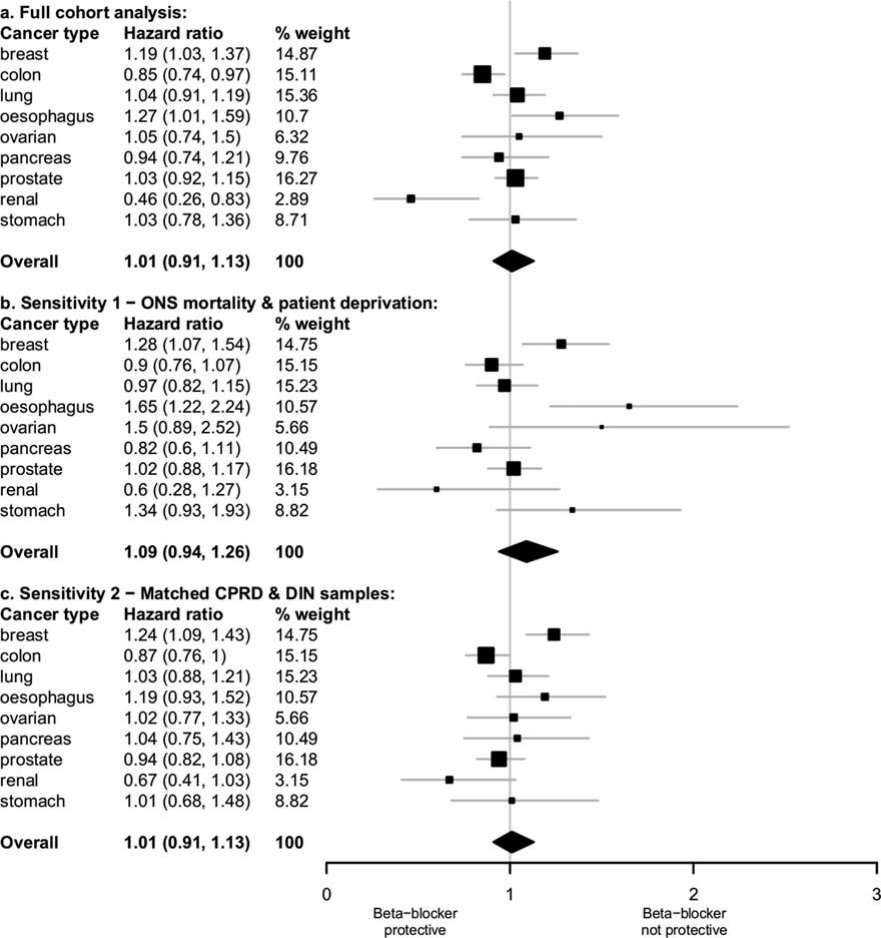
HRs of survival for patients prescribed β-blocker therapy compared with patients prescribed other blood pressure lowering medicines.

There was no difference in adjusted mortality rates between patients in the CPRD receiving β-blockers (with or without other BPLM) and those on other BPLMs only (overall HR=1.01, 95%CI 0.91 to 1.13; table 3; figure 1). This compares to a small but statistically significant impact of β-blockers on mortality in DIN (HR=1.18, 95%CI 1.04 to 1.33). However, the Wald test directly comparing these HRs was not statistically significant, although it did approach significance (p=0.063).

**Table 3:** Comparison of patients using β-blockers versus patients using other BPLMs only from the CPRD and DIN studies: pooled HRs (95% CI) from meta-analyses of cancer site-specific results

Comparison (vs controls)	CPRD	DIN	p Value†
All patients using β-blockers	1.01 (0.91 to 1.13)	1.18 (1.04 to 1.33)**	0.06
Patients using β-blockers only	0.94 (0.82 to 1.07)	1.37 (1.16 to 1.61)***	<0.001***
β-Blockers and other BPLM	1.06 (0.95 to 1.19)	1.11 (0.91 to 1.34)	0.69
Non-selective β-blockers only	0.96 (0.8 to 1.15)	1.21 (0.94 to 1.55)	0.14

**p<0.01; ***p<0.001.

†Wald tests of CPRD vs DIN HRs.

BPLMs, blood pressure lowering medicines; CPRD, Clinical Practice Research Datalink; DIN, Doctors’ Independent Network.

For the subset of patients on β-blockers alone, we again found no significant impact on cancer mortality (HR=0.95, 95% CI 0.83 to 1.09), as opposed to a significant effect (HR=1.37, 95% CI 1.16 to 1.61) reported by Shah *et al*.^22^ In this instance the comparison between studies was significant (p<0.001). For the remaining two subsets, of patients on β-blockers plus other BPLM and patients on non-selective β-blockers, the two studies returned similar non-significant results.

Mortality for individual cancer sites (table 4).

**Table 4:** Cancer site-specific and pooled HRs (95% CI) from CPRD and DIN studies for all patients using β-blockers versus patients using other BPLMs only

Cancer site	CPRD full cohort	CPRD sensitivity analysis 1: ONS mortality and deprivation	CPRD sensitivity analysis 2: Matched CPRD and DIN samples	DIN primary analysis	p Value†
Sample size (on β-blockers; controls)	4030; 7272	2528; 4514	4105‡; 7197‡	1406; 2056	–
Cancer sites					
Breast	1.19 (1.03 to 1.37)*	1.28 (1.07 to 1.32)***	1.24 (1.09 to 1.43)**	1.09 (0.80 to 1.49)	0.62 (0.46)
Colon	0.85 (0.74 to 0.97)*	0.9 (0.76 to 1.07)	0.87 (0.76 to 1.0)*	1.00 (0.77 to 1.30)	0.28 (0.36)
Lung	1.04 (0.91 to 1.19)	0.97 (0.82 to 1.15)	1.03 (0.88 to 1.21)	1.12 (0.89 to 1.41)	0.59 (0.56)
Oesophagus	1.27 (1.01 to 1.59)*	1.65 (1.22 to 2.24)***	1.19 (0.93 to 1.52)	1.05 (0.69 to 1.60)	0.44 (0.61)
Ovarian	1.05 (0.74 to 1.5)	1.5 (0.89 to 2.52)	1.02 (0.77 to 1.33)	1.14 (0.63 to 2.06)	0.82 (0.74)
Pancreas	0.94 (0.74 to 1.21)	0.82 (0.6 to 1.11)	1.04 (0.75 to 1.43)	1.88 (1.09 to 3.25)*	0.023* (0.069)
Prostate	1.03 (0.92 to 1.15)	1.02 (0.88 to 1.17)	0.94 (0.82 to 1.08)	1.54 (1.13 to 2.09)**	0.016* (0.004)**
Renal	0.46 (0.26 to 0.83)**	0.6 (0.28 to 1.27)	0.67 (0.41 to 1.03)	1.14 (0.52 to 2.52)	0.069 (0.25)
Stomach	1.03 (0.78 to 1.36)	1.34 (0.93 to 1.93)	1.01 (0.68 to 1.48)	1.44 (0.76 to 2.74)	0.35 (0.35)
All patients using β-blockers	1.01 (0.91 to 1.13)	1.09 (0.94 to 1.26)	1.01 (0.91 to 1.13)	1.18 (1.04 to 1.33)**	0.063 (0.063)

*p<0.05; **p<0.01; ***p<0.001.

†Wald tests of CPRD full cohort versus DIN (CPRD matched sample vs DIN).

‡Median across bootstrap samples.

BPLMs, blood pressure lowering medicines; CPRD, Clinical Practice Research Datalink; DIN, Doctors’ Independent Network; ONS, Office for National Statistics.

Using CPRD, mortality rates for patients receiving β-blockers, compared to those on other BPLMs only, were significantly higher for breast cancer (HR=1.19, 95% CI 1.03 to 1.37) and oesophageal cancer (HR=1.27, 95% CI 1.01 to 1.59) but significantly lower for patients with colon (HR=0.85, 95% CI 0.74 to 0.97) and renal cancer (HR 0.46, 95% CI 0.26 to 0.83), with no significant differences for other cancer sites. Using DIN, Shah *et al*^22^ reported survival to be significantly poorer for patients with pancreas and prostate cancer, with no other differences. Thus, for four of the nine cancer sites, our CPRD study found a significant association of mortality with β-blockers, whereas the DIN study did not; for two other sites, this association was reversed.

Direct comparison of the cancer site-specific HRs from the two studies using Wald tests found no significant differences except for pancreatic cancer (p=0.023) and prostate cancer (p=0.016). For both cancers, CPRD returned HRs close to 1, whereas DIN produced much higher values. There was also significant heterogeneity of treatment effect across cancer sites in CPRD (p=0.004) in contrast to non-significant heterogeneity in DIN (p=0.41).

### Results of sensitivity analysis

Sensitivity analysis using the subset of CPRD practices for which ONS mortality and patient-level IMD scores were available produced little change in the overall HR for death associated with β-blockers (table 4: HR=1.09, 95% CI 0.94 to 1.26). HRs for individual cancer sites likewise did not change greatly, although those for colon and renal cancers ceased to be statistically significant, at least partly due to the reduced sample.

Analysis of the CPRD sample(s) selected to match Shah *et al*^22^ DIN cohort resulted in an overall HR and 95% CI identical to our primary analysis, and only small changes in the results for individual cancer sites, although the HRs for oesophageal and renal cancers ceased to be statistically significant (table 4), as did the direct comparison of the CPRD and DIN HRs for pancreatic cancers (p=0.069). Repeating our analyses by adjusting for clustering of patients within practices and excluding patients who survived for less than a year made no substantive difference to any of the results (see online supplementary table S3).

## Discussion

We conducted a fully independent, external replication of a study based on one PCD using data from an alternative database. Our replication used the CPRD, a larger data set than the DIN; hence, our total patient sample was more than three times the size of the original study. As far as possible, we sampled from the same patient population and used identical methods to the original study so as to minimise any sources of variation other than the database itself.

Using CPRD we found no evidence for an association between β-blocker use and cancer mortality, either in the full CPRD cohort or for patients on β-blockers only—where the strongest effect was observed by Shah *et al*^22^ using DIN. Results for individual cancer types also differed considerably, indicating an entirely different set of statistically significant cancer sites. However, most study differences disappeared under direct comparison of the HR estimates, with only the treatment effects for patients with pancreatic and prostate cancers, and for those on β-blockers alone remaining significantly different—for whom β-blocker use was associated with mortality in DIN but not in CPRD. Thus, with these exceptions, all treatment effect estimates from the two studies agreed within the range of random variation. These results were unchanged in all essentials under sensitivity analyses using CPRD subsamples, with linked ONS mortality and patient-level deprivation measures, and matched with DIN on cancer prevalence rates and other sample characteristics.

It is informative to compare both of these studies to a series of investigations by a group working at Queen's University, Belfast, who also used CPRD to investigate the effects of β-blocker usage on mortality from breast,^27^ colon^38^ and prostate cancer^39^ by using a methodology that differed in a number of respects. No significant associations were found for any of these cancer sites, in contrast to both our study (breast, colon) and to Shah *et al*^22^ study (prostate). However, the CIs reported by all three teams overlapped considerably—with the exception of prostate cancer from Shah *et al*^22^ study (see online supplementary table S4)—indicating that all treatment effect estimates were equivalent within statistical limits of accuracy.

Unresolved differences between CPRD and DIN, after direct comparison, were few and mainly low level, but it is worthwhile to consider why these should remain. Results did show some sensitivity to database differences in patient demographics, though not enough to explain all the discrepancies in the results. The very different clinical computing systems used may have affected aspects of recorded care—possibly more than any practice or sample characteristic^40^—but the small number of study differences suggests that any overall impact was minimal, though an influence on specific data items and results is plausible. The much lower mortality rate in DIN suggests that death may have been defined differently or recorded less reliably. However, for this to explain the difference in effect estimates, the act of recording mortality in DIN would have to be associated with prescription of β-blockers and not with other BPLMs, and also with certain types of cancer but not others, which seems unlikely.

The unresolved differences might simply be statistical artefacts. Unmeasured confounding factors could vary in distribution between the data sets. Also, all the discrepant results concerned largely exploratory subgroup analyses done within a framework of multiple significance testing and arguably, an α-level higher than 5% would be more appropriate: at α=1%, the only unresolved difference is for patients on β-blockers alone. The QResearch replication study,^20^ likewise, identified a number of within-study and between-study discrepancies in subgroup analyses relating to different statin compounds; in all three of the Belfast group's studies, despite no overall associations, subgroup analyses found significant relationships between cancer survival and one to three specific β-blocker compounds, though not always the same compound and not always in the same direction.^27^ ^38^ ^39^ The large scale of many EHRs may encourage researchers to undertake multiple subgroup analyses without any firm hypotheses, and may also foster the idea that size alone offers some protection against incorrect inference; yet the rate at which inconsistent results occur in EHR-based studies strongly suggests that issues of multiple testing, ‘fishing’ for results, and spurious significance apply as much to these data sources as they do to much smaller data sets, possibly even more so given the potential for bias from residual and uncontrolled confounding.

The results of our study, therefore, present a somewhat complex picture: examined separately and purely in terms of statistical significance, the CPRD-based and DIN-based studies provided rather different pictures of the risks of β-blockers overall and in relation to different cancer types. The survival disadvantage observed by Shah *et al*^22^ was not insubstantial: an increased point risk of death of 18%, increasing to 37% for patients on β-blockers only. These results were not present in our replication study, including across a variety of sensitivity analyses. Yet when directly compared, with the main exception of the β-blocker, only subgroup estimates of treatment effect from the two studies did not differ statistically. Drawing a satisfactory conclusion from these findings is not easy. Focusing on the direct statistical comparisons of the study effect estimates, this study taken in combination with our previous replication study and other non-independent replications, suggests that the application of identical analytical methods to different UK PCDs yields treatment effect estimates that are usually comparable within statistical limits of accuracy. Nevertheless, when taken separately, our study and that of Shah *et al*^22^ point to very different conclusions about the safety of β-blockers in this patient population; this indicates that single studies, even when demonstrating notable effects based on well-substantiated databases, do not guarantee generalisable results.

### Limitations

Differences between the DIN and CPRD databases meant that while we were able to exactly replicate the great majority of the components of the original study, there were a few exceptions. The data sets may have differed in their definitions of all-cause mortality, as each use their own bespoke algorithm. For area deprivation, Shah *et al*^22^ used 2004 IMD scores in national quintiles based on each patient's postcode. Equivalent scores were only available to us for a subset of CPRD; so we used 2004 practice-postcode IMD scores, obtained for all practices, from the CPRD organisation as a linked data set. We tested for the impact of these factors by running a sensitivity analysis using the subset of CPRD patients for which linked ONS data on the date of death and residential IMD 2004 scores were available. In all other respects, this study replicated the original with respect to the population, and variable definitions and methods of analysis.

The overall raw mortality rate in our CPRD cohort was substantially higher than in the DIN cohort and a much higher proportion of CPRD cancers were that of the prostate. Patients in the CPRD cohort were also likely to have been diagnosed more recently, to live in areas of higher deprivation and to be male. However, analysis of subsets of the CPRD cohort matched to DIN did not account for the difference in overall mortality rates nor did it substantially alter our findings. Neither the complete details of how Shah *et al*^22^ defined mortality nor the CPRD mortality algorithm were available to us; thus, our ability to uncover the reasons for these different mortality rates was limited. However, sensitivity analysis using ONS official mortality data suggested that the CPRD mortality rates at least are robust.

We intentionally did not try to improve on the analysis methods used by Shah *et al*,^22^ even though these have received some criticism,^32^ ^38^ since for our purpose it was important to keep the analysis methods constant. Criticisms include: not linking to cancer registries; lack of control for stage of disease or treatment; not differentiating β-blocker use prior to cancer and postcancer diagnosis; and use of patients on other antihypertensives as the comparator. Most of these criticisms were in fact discussed by Shah *et al*^22^ in their paper and justified there as part of the methodology. Importantly, the Belfast group's CPRD-based studies took account of most of these issues and still yielded effect estimates very similar to our own.

### Conclusion

This replication of one UK PCD-based study in a second completely independent PCD, using the same methods and sampling the same population, has revealed a complex pattern of similarities and differences in both the makeup of the patient cohorts and in the findings from analysis. When directly compared, with the exception of certain subgroup results, estimates of treatment effect did not differ statistically and in this sense, this study adds to previous replication work in finding that when analysed to a common protocol, different UK PCDs produce treatment effect estimates that generally agree within statistical tolerance. Nevertheless, considered separately, this study and the original DIN-based investigation point to very different conclusions regarding the safety of β-blockers for solid patients with cancer. Hence, our results also show that single studies based on even these internally well-validated databases may not guarantee generalisable results. Therefore, great care must be taken in drawing any firm conclusions, particularly where subgroup results are concerned. In all cases, confirmatory studies using at least one other independent data source are strongly recommended.
